# Association of Zinc, Copper and Magnesium with bone mineral density in Iranian postmenopausal women – a case control study

**DOI:** 10.1186/2251-6581-13-43

**Published:** 2014-03-06

**Authors:** Rezvan Razmandeh, Ensieh Nasli-Esfahani, Reza Heydarpour, Farnoush Faridbod, Mohammad Reza Ganjali, Parviz Norouzi, Bagher Larijani, Davood Khoda-amorzideh

**Affiliations:** 1 Diabetes Researcher Center, Endocrinology and Metabolism Clinical Sciences Institute, Tehran University of Medical Sciences, Tehran, Iran; 2 Endocrinology and Metabolism Research Center, Endocrinology and Metabolism Research Institute, Tehran University of Medical Sciences, Tehran, Iran; 3 Center of Excellence in Electrochemistry, University of Tehran, Tehran, Iran; 4 Rajaie Cardiovascular Medical and Research Center, Iran University of Medical Sciences, Tehran, Iran

**Keywords:** Trace elements, Osteoporosis, Postmenopausal women

## Abstract

**Background:**

The risk of inadequate nutrition such as trace elements and vitamin deficiencies is considerable in postmenopausal women. The aim of this study was to compare trace elements (Zinc, Copper and Magnesium) concentration in nail, urine and serum among osteoporotic postmenopausal women with control group in Iran.

**Methods:**

Forty eight postmenopausal women aged 36–60 years, were recruited, consisting 30 osteoporotic patients and 18 healthy controls. Blood, nail and urine concentration of Zinc (Zn), copper (Cu) and magnesium (Mg) were determined using Inductively Coupled Plasma -Atomic Emission Spectrometry (ICP-AES) method. Their Bone Mineral Density was measured by Dual X-ray Absorption (DEXA) method.

**Results:**

The urine level of trace elements had significant difference between osteoporotic groups and controls (p < 0.001). Moreover Mg level significantly differed in serum between two groups (p < 0.04). There was no statistically significant difference in trace minerals in nail beyond groups.

**Conclusion:**

Our findings indicate that Urine Zn level could be considerable an appropriate marker for bone absorption, usage of Zn supplements in postmenopausal women may result a beneficial reduction in osteoporotic risk.

## Background

Osteoporosis is the most common metabolic bone disease [1]. It is characterized by low bone mass and micro-architectural deterioration of bone tissue, leading to enhanced bone fragility and a consequent increase in fracture risk [2]. Osteoporosis is a multi factorial disease and several factors such as, genetics, gender, age, race, weight, medical conditions, medication and life style risk factors are considered to be important determinants of it [3]. The Iranian Multicenter Osteoporosis Study (IMOS) estimated that the prevalence of osteoporosis among women older than 50 years is 6 percent, which is less than other countries such as Canada and Japan [4].

One of the most important modifiable factors in the development and maintenance of bone mass is nutrition [5]. Adequate nutrition plays a major role in the prevention and treatment of osteoporosis [6]. In recent years, there has been a resurgence of interest in studies concerning the role of elements in the development and maintenance of the skeleton [7].

Zinc (Zn) is an essential mineral that is a component of more than 200 enzymes and is known as to be necessary for normal collagen synthesis and mineralization of bone [8]. Copper (Cu), a cofactor for lysyl oxidase, is required in the cross-linking of collagen and elation. Cu deficiency causes inhibition of bone growth and osteoporosis [9]. The other element, Magnesium (Mg) appears to be important in bone cell activity. It is shown to be mitogenic for osteoblasts and its depletion causes cellular growth inhibition, in vitro [10].

The growths of nails, and their matrix components, are influenced by several physiological, pathological, and environmental factors [11]. Because of the slow rate of nail growth, the elemental composition of the nail is also expected to be affected by transient factors controlling serum components [12]. The mineral components of nail clippings may therefore reflect the long-term patterns of mineral metabolism such as Hypercreatinemia, Hyperthyroidism and Iron Deficiency Anemia [13,14].

To understand the status of elements on postmenopausal women with osteoporosis, we have investigated the Zn, Cu and Mg Cu levels in postmenopausal women with osteoporosis and without osteoporosis.

## Method

In this case–control study (May 2008 to may 2009) forty eight postmenopausal women aged 36–60 years, according to Bone Mineral Density (BMD) divided into two groups 30 Postmenopausal Osteoporotic women (case) (T score > -2.5) and 18 non-Osteoporotic postmenopausal women (control) (T score < -1.0) [11]. They were recruited among patients who applied to outpatient osteoporosis clinic of Tehran University of Medical Sciences (Dr Shariati Hospital). All the postmenopausal women who had passed one year of their last menstrual period were included in the study. Exclusion criteria were as follows:

1. Arthritis rheumatoid, Diabetes mellitus, Systemic lupus erythematosus, Hypo or hyperthyroidism, Hypo or hyperparathyroidism, Hepatic failure, Renal failure, Cirrhosis, Cushing’s syndrome, Adrenal failure, Cancers.

2. Menstrual disorders as initial menstrual cycle after 18 and permanent discontinuation before 40 years old.

3. Smoking (more than half a packet per day), addiction and the history of alcohol consumption for more than 5 years.

4. Professional sports, past history of lumbar fractures, fractures because of simple falls, spinal deformity, and seeking admission in the last two weeks or complete best rest for 3 consecutive months.

5. Previous Usage of Estrogen Progesterone, Furosemide, Antiepileptic drugs, Corticosteroids, Heparin, Thiazide and any trace mineral Supplements.

The study was performed in accordance with the Declaration of Helsinki and subsequent revisions and approved by ethics committee at Endocrinology and Metabolism Research Centre (EMRC). Informed consent was sought and obtained from individuals before enrollment into the study.

Having received the letters of consent, the related questionnaires were completed and clinical examinations such as height and weight were carried out. The weight of all participants with minimum of clothes by using digital scales (Seca) with an accuracy of 0.1 (kg) was measured. Height was measured using a wall stadiometer (precision 5 mm) with the patient standing upright and without shoes. The body mass index (BMI) was calculated by dividing subject’s weight (kg) by the square of their height (m^2^). The baseline information of food intake was collected by using a 24*-*hour recall method on three consecutive days.

BMD was measured by DXA using Lunar DPX-MD device (Lunar Corporation, Madison, Wisconsin, 53713. USA). The DXA device was calibrated daily and weekly by using appropriated phantoms methods. To assess BMD, second to fourth lumbar spine and from the femur bone (neck, trochanter and the whole femur), bone density was calculated based on gr/cm^2^.

Blood and morning second void urine samples were collected after an overnight fast; precautions were taken to avoid contamination. Serum PTH, Vit D, Mg, Zn, Cu, ALT, AST, Alb, Cr, Ca, P, and TSH and urine, Zn, Cu and Mg were collected in the metal free plastic tubes. Toe nail clippings from all 10 toes were collected within 8 weeks of inclusion in the study and were stored in small plastic bags at room temperature. Solid sample of nail was washed immediately with distilled water and then alcohol dried and stored. The sufficient amounts of nail samples were weighed carefully and transfer in to a beaker and added 10 ml of concentrated nitric acid. Then, the solutions were heated for complete digestion. The resulted solution diluted in a volumetric flask with distilled water. Certain amount of blood and urine samples were also mixed with concentrated nitric acid to remove interfering of organic compounds, the resulted solution were then centrifuged.

Inductively Coupled Plasma Atomic Emission Spectrometer (ICP-AES) has become the most appropriate technique for trace element determination [12]. A Varian ICP-AES (model: VISTA-MPX) was used for analysis. The amount of trace elements of each samples (serum, urine and nail) were measured by ICP-AES instrument using calibration method. This method was based on special software to calculate the numerical calculation analyze signal and reduce noise. In current method all the measurement done by continues cycle voltmeter. This means that in specified temperature range of Cyclic voltammeter (CV) wave potential is applied to electrode and provided number frequency that can shows any changes in flow produced with electrode.

There are no any cutoff for trace elements concentration in Iran, therefore we used the cutoffs’ which was determined in similar articles. Cutoff for plasma Zn was 75–120 microgram/dl, and for plasma Cu and plasma Mg were considered 70–140 and 19.5-2.33 microgram/dl respectively. Serum chemical estimations were performed using enzymatic method (BioLif 24i, Premium, Tokyo, Boeki medical system, Japan) and Radioimmunoassay for Vit D (IDS kits, UK), PTH (Diasorin kits, USA) and TSH (Automatic Gama Counter, Wizard of Swiss).

The measurement of Zn, Cu and Mg in diets was done by modified software, Food Processing 2 (FP2).

The significance of difference in trace elements level in samples between two groups was tested using independent *t*-test analysis. Association between variables was determined using the Pearson’s correlation analysis on Microsoft excel and SPSS software version 13.0 (California Inc.). A two sided P-value <0.05 was considered statistically significant for the *t*-test and Pearson correlation analysis.

## Result

Table 1 shows the baseline characteristics and biochemical findings of two groups based on BMD. There was no statistically significant difference between two groups in the characteristics.

**Table 1:** Comparison of baseline characteristics between osteoporotic and control groups

**Variable**	**Osteoporotic group**	**Control group**	**p- value**
	**Mean (SD)**	**Mean (SD)**	
**Age [years]**	58.4 (6.4)	57.6 (4.7)	NS
**BMI [kg/m**^**2**^**]**	28.4 (4.5)	27.7 (4.3)	NS
**Menopausal age [years]**	51.6 (2.4)	50.8 (3.2)	NS
**AST [Iu/l]**	22.86 (9.86)	28.18 (22.14)	NS
**ALT(Iu/l)**	11.90 (5.67)	15.56 (13.08)	NS
**Alp [Iu/l]**	176.03(115.20)	187.94 (130.10)	NS
**Ca [mg/dl]**	8.7 (0.38)	9.09 (0.39)	NS
**P [mg/dl]**	3.76 (0.40)	3.95 (0.59)	NS
**Vit D [ng/mg]**	**29.89 (31.70)**	12.56 (14.99)	P = 0.04
**PTH [pg/ml]**	65.57 (35.91)	69.43 (24.55)	NS
**Alb [mg/dl]**	5.01(0.31)	4.90 (0.38)	NS
**Cr [mg/dl]**	0.91(0.21)	0.94 (0.10)	NS

*BMI* body mass index, *PTH* paratormone hormone, *Alb* albumin, *P* phosphorus, *Ca* calcium, *ALT* alanine amino transpherase, *AST* aspartate amino transpherase, *Vit* vitamine, *Alb* albumin, *Alp* alkaline phosphatase. The level of vitamin D in osteoporotic group was higher than control group.

Table 2 shows the levels of trace elements in serum, urine and nail in two groups. The serum Mg level was significantly less in cases (P = 0.04) than in controls. The urine Zn level between osteoporotic and control group was significantly different (P < 0.001).

**Table 2:** Comparison of Cu, Mg, and Zn in plasma, urine, and nail between osteoporotic and control groups

**Trace elements**		**Control group**	**Osteoporotic**	**P - value**
		**Mean (SD)**	**Mean (SD)**	
Serum	**Cu [ppm]**	1.12 (0.53)	0.98 (0.43)	NS
	**Mg [ppm]**	19.80 (8.23)	15.08 (4.89)	P = 0.04
	**Zn [ppm]**	0.84 (0.43)	0.68 (0.23)	NS
Nail	**Cu [ppm]**	7.84 (10.71)	6.91(8.15)	NS
	**Mg [ppm]**	87.88 (78.21)	90.76 (97.30)	NS
	**Zn [ppm]**	81.60 (86.14)	82.57 (103.07)	NS
Urine	**Cu [μg/g]**	0.049 (0.013)	0.038 (0.024)	NS
	**Mg [μg/g]**	5.80 (1.36)	6.56 (2.24)	NS
	Zn [μg/g]	0.51(0.35)	1.72 (1.49)	P< 0.001

The significant positive correlation between urine Cu and serum TSH (p = 0.02 r =0.37). There was significantly negative correlation between PTH and urine Zn (p = 0.00, r = -0.53), (p = 0.01, r = -0.47).

There was no correlation between case and control groups in the nails’ samples.

## Discussion

Some studies have shown the effect of nutrition on bone turnover and osteoporosis. In this study we investigate Zn, Cu and Mg in serum, urine and content of nail in post menopausal women that referred to clinic of osteoporosis - Shariati Hospital in Tehran University and divided them in two groups of osteoporotic and non -osteoporotic women.

Zn is involved in bone metabolism is based on observations that osteoporotic patients have increased urinary Zn losses [15]. Urine Zn excretion in osteoporotic patients was significantly lower than control group and there was not any meaningful difference in serum Zn in two groups. Actually bone demineralization in osteoporosis could cause high excretion of trace minerals, although in study group the level of Zn in plasma was higher than control group in our study. Similar to our study, Reginster et al. [16] reported no significant difference in Zn and Cu plasma levels between postmenopausal women with osteoporosis and non-osteoporotic subjects. Relea et al. [8] have reported that the urine clearance of Zn in postmenopausal women was higher than in the normal women. In contrast, several studies found that serum Zn and Cu levels were lower among patients with postmenopausal osteoporosis than controls [17-19]. A relationship exists between the excretion of Zn in 24-hour urine and the appearance of osteoporosis, and the urinary Zn/creatinine relationship is a factor to take in mind in the osteoporosis [20,21].

Regarding the relationship between serum [22-24] and urine Zn, it seems that higher serum PTH in osteoporotic group comparing to normal amounts of PTH, rising Zn urinary excretion. The lack of significant difference in serum Zn between case and control groups can be attributed to higher than normal PTH concentration. In present study in consistency with other studies, urinary Zn levels in control group were higher than cases.

The indicators of discrepancy and accuracy of Zincuria are highly indicative of the value of this parameter as biochemical markers of bone resorption for the diagnosis of osteoporosis [15,20].

We found that there is no significant difference in serum level of Zn in two groups and it may be because of having small population size. Several studies reported that serum Zn and Cu levels were lower among patients with postmenopausal osteoporosis than controls [25-27]. In contrast, Reginster et al. [16] significant difference in Zn and Cu plasma levels between two groups. Two studies [28,29] found that serum Zn level was lower among the postmenopausal osteoporotic women than controls.

We found similar Cu levels in osteoporotic and non-osteoporotic postmenopausal women. Other studies [13,14] have revealed that Cu deficiency resulted in postmenopausal osteoporosis. In a study by Conlan et al. [15] elderly patients with femoral neck fractures were found to have significantly lower serum Cu levels than controls adjusted for age and sex. Although the Cu content of bone was negatively correlated with bone Ca, bone density and collagen content in aging mice [16], bone Cu levels in human subjects with osteoporosis were found to be the same or slightly higher than normal individuals of the same age [10,17].

Tranquilli et al. [30] reported that the uptake of Mg, P, and Cu are low in women with postmenopausal osteoporosis as compared to the control group and that this correlated with bone mineral content. Cross-sectional studies demonstrated a strong correlation between low BMD, low dietary Ca intake, and serum Cu levels in postmenopausal women [19].

Our study showed that the contents of serum Mg in osteoporotic patients is significantly lower than control group. Mg appears to be important in bone cell activity and contributes macro element quantities to bone ash and is essential for appropriate calcium metabolism, affecting calcium balance. Trabecular but not cortical bone was improved with per oral Mg in a small group of postmenopausal osteoporotic women [31,32].

Carpenter [33] demonstrated that Mg deficiency has an effect on osteocalcin synthesis and secretion and that this resulted in decreased osteocalcin synthesis. Two studies [20,21] reported that serum Mg in osteoporotic women were significantly lower than the value in matched normal subjects. Conversely, Steidl et al. [17] found a significant decrease in erythrocyte Mg content in patients with postmenopausal and senile osteoporosis but not in their serum levels.

Gur et al. [34] reported that Mg levels in serum were lower among patients with postmenopausal osteoporosis than the controls.

The level of Cu and Mg secretion in urine did not have any significant difference in two groups. Urine is a clinical specimen often used in medical diagnostics for monitoring of elements concentrations and osteoporosis. Basing on Dlugaszek study, they concluded that BMD and age of postmenopausal osteoporosis may impact greatly on the elemental status of 24-h urine [35]. The other research showed that Mg levels in 24-hour urinary excretion of Mg were increased in the postmenopausal women compared with the premenopausal women and postmenopausal hypermagnesiuria probably originates from increased intestinal magnesium absorption [36].

Schlemmer reported [37] that Ca supplementation influences the urinary excretion of Mg after menopause. Two studies [38,39] found that women with osteoporosis displayed Mg deficiency and a highly significant correlation existed between increased Mg urinary excretion and low serum Ca.

There are a little evidence related to association between the contents of trace elements in nail and bone densitometry in postmenopausal women.

In our research there was no correlation between case and control groups in the nail samples.

Concentration of Zn in fingernails was significantly but negatively correlated with bone mineral density and a significant positive correlation of Ca/Zn ratio of fingernails was observed with bone mineral density. The content of Zn in nails may be affected by alterations in bone Zn status and a potential application of nail Zn level or Ca/Zn ratio as an indicator of postmenopausal and senile osteoporosis.

Three studies [25-27] in postmenopausal women have been shown lower nail Ca concentrations than in premenopausal women. Their BMD status showed a significant positive correlation with nail Ca content. The demonstration of an inverse relationship between nail Ca and Mg content may contribute to the understanding of the role of these minerals in the development of osteoporosis [28].

An Irish study demonstrated that nail testing as such a method is inconclusive but these preliminary findings suggest that changes in bone proteins seen in osteoporosis may be mirrored by changes related to structural proteins [40,41].

In Table 1 level of vitamin D in osteoporotic group was higher than control group.

We had limitation in our study: 1. this study is a single center study in osteoporosis clinic of Tehran University of Medical Sciences (Dr Shariati Hospital) 1.not all of our studied patients comes in repeated follow ups, 2. Delay in result of laboratory tests 3. It’s difficult to find the suitable samples.

Some studies have shown that all patients with osteoporosis treated with vitamin D and calcium are serum levels of calcium and vitamin D than their peers in non-osteoporotic, as shown in our study [5,40].

## Conclusion

Our findings indicate that Urine Zn level could be considerable an appropriate marker for bone absorption, usage of Zn supplements in postmenopausal women may result a beneficial reduction in osteoporotic risk.

## Abbreviations

EMRI: Endocrinology and Metabolism Research Institute; TUMS: Tehran University of Medical Sciences; BMD: Bone mineral density; ICP-AES: Inductively coupled plasma -atomic emission spectrometry; BMI: Body mass index; DXA: Dual x-ray absorption; Zn: Zinc; Cu: Copper; Mg: Magnesium; AST: Aspartate aminotransferase; ALT: Alanine aminotransferase; Alp: Alkaline Phosphatase; Ca: Calcium; Vit D: Vitamin D; TSH: Thyroid - stimulating hormone; PTH: Parathyroid hormone; Alb: Albumin; Cr: Creatinine; P: Phosphorus.

## Competing interests

The authors declare that they have no competing interests.

## Authors’ contributions

Conception and study design and coordination: RH, Drafting manuscript and Data analysis: RR, Participation in its sequence alignment: DK-A, Review of manuscript and important intellectual content: EN-E, All authors read and approved the final manuscript.
